# Incarcerated Umbilical Hernia Following Therapeutic Paracentesis in a Cirrhotic Patient

**DOI:** 10.7759/cureus.23851

**Published:** 2022-04-05

**Authors:** Sohaib Khatib, Taher Sabobeh, Mohamed Ahmed, Khalid Abdalla, Erin Algeo

**Affiliations:** 1 Internal Medicine, University of Missouri, Kansas City School of Medicine, Kansas City, USA

**Keywords:** abdominal paracentesis, paracentesis, cirrhosis, incarceration, umbilical hernia

## Abstract

Umbilical hernia is a relatively common complication developing in patients with liver cirrhosis with recurrent ascites. Abdominal paracentesis is considered the mainstay procedure to manage refractory ascites and to diagnose spontaneous bacterial peritonitis. Incarceration of umbilical hernia is a rare but serious adverse event following therapeutic paracentesis that requires prompt management. We describe a case of an incarcerated umbilical hernia following paracentesis requiring surgical repair in a cirrhotic patient.

## Introduction

Umbilical hernias are relatively common surgical abnormalities of the abdominal wall with about 175,000 umbilical hernia repairs performed yearly in the United States [1]. Umbilical hernias in adults result from acquired weakness of the umbilical ring which leads to herniation of abdominal contents [2]. Umbilical hernias can be potentially complicated by incarceration or strangulation with mortality estimates ranging from 3% to 14% [3]. 

Abdominal paracentesis is generally a safe procedure that allows the analysis of ascetic fluid to identify the causes of ascites and also represents the treatment of choice for large volume ascites with marked abdominal discomfort [4]. Rare complications of this procedure include bleeding or intestinal perforation. In our case, we report a cirrhotic patient with an umbilical hernia due to recurrent ascites who developed umbilical hernia incarceration after therapeutic paracentesis. 

## Case presentation

A 76-year-old African American male presented to the emergency department with hematuria and abdominal pain. He has a previous past history of advanced alcoholic liver cirrhosis complicated by recurrent ascites, hypertension, cerebrovascular accident, diabetes mellitus type II, and paroxysmal atrial fibrillation. The patient had a recent hospitalization with alcoholic hepatitis, acute kidney injury, and hematuria presumed secondary to a lower urinary tract infection with extended-spectrum beta-lactamase-producing *E. Coli*. He was sent home on appropriate intravenous antibiotics and planned for outpatient cystoscopy. 

On initial encounter, physical examination was remarkable for abdominal distension with positive fluid shifting dullness, non-tender umbilical hernia with no palpable content, and lower abdominal tenderness. Vital signs included normal blood pressure at 110/70, normal heart rate of 74, and normal temperature of 98.3 F. Initial laboratory investigations included: a WBC (white blood cell count) of 6.25 TH/uL, hemoglobin of 8.5 g/dL, platelets of 161 TH/uL, with a normal kidney function, liver function, and coagulation panel (Table 1).

**Table 1:** Laboratory values including CBC (complete blood count), BMP (basic metabolic profile), LFTs (liver function tests), coagulation studies, and peritoneal fluid analysis Laboratory values including CBC (complete blood count), BMP (basic metabolic profile), LFTs (liver function tests), coagulation studies, and peritoneal fluid analysis

Laboratory value	Result	Reference range
WBC (White blood cell count)	6.25 TH/uL	4.5-11 TH/uL
Hemoglobin	8.5 g/dL	14-18 g/dL
MCV (Mean corpuscular volume)	98 fL	80-100 fL
Platelets count	161 TH/uL	150-450 TH/uL
Sodium	141 mmol/L	135-145 mmol/L
BUN (Blood urea nitrogen)	14 mg/dL	6-24 mg/dL
Creatinine	0.80 mg/dL	0.7-1.3 mg/dL
Calcium	7.9 mg/dL	8.6-10.3 mg/dL
Protein	5.8 g/dL	5.5-8.3 g/dL
Albumin	2.6 g/dL	3.4-5.4 g/dL
AST (Aspartate aminotransferase)	36 U/L	8-33 U/L
ALT (Alanine transaminase)	15 U/L	4-36 U/L
Bilirubin total	0.4 mg/dL	0.1-1.2 mg/dL
PT (Prothrombin time)	14.3 Seconds	11-13.5 Seconds
INR (International normalized ratio)	1.1	0.8-1.1
Peritoneal fluid RBC	1000 /uL	None
Peritoneal fluid WBC	57 /uL	Less than 300 /uL
Peritoneal fluid neutrophils%	5%	Less than 250 PMNs

**Figure 1:**
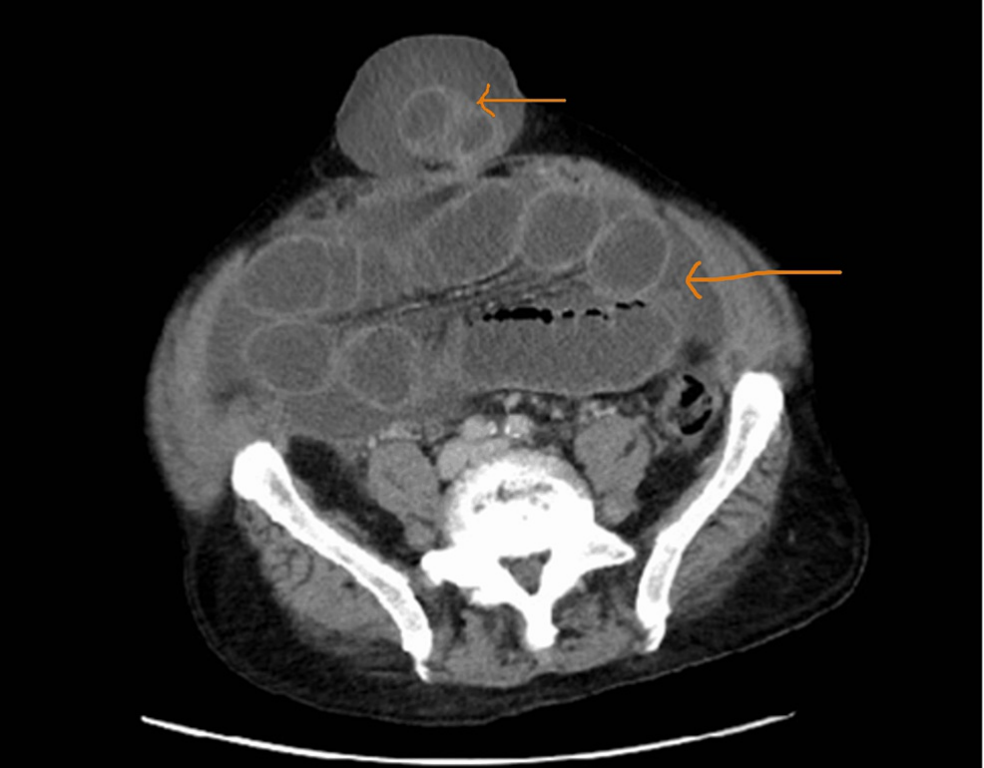
CT scan of the abdomen without contrast on admission prior to abdominal paracentesis CT scan of the abdomen without contrast on admission prior to abdominal paracentesis, showing abdominal ascites (orange arrow), and umbilical hernia with no contents (red arrow).

Urine analysis showed > 40 count RBC and WBC with a positive leukocyte esterase. CT scans of the abdomen and pelvis were only remarkable for large volume abdominopelvic ascites, colonic diverticulosis, and liver cirrhosis changes (Figure 1). An umbilical hernia without content was noted on the scan. 

**Figure 2:**
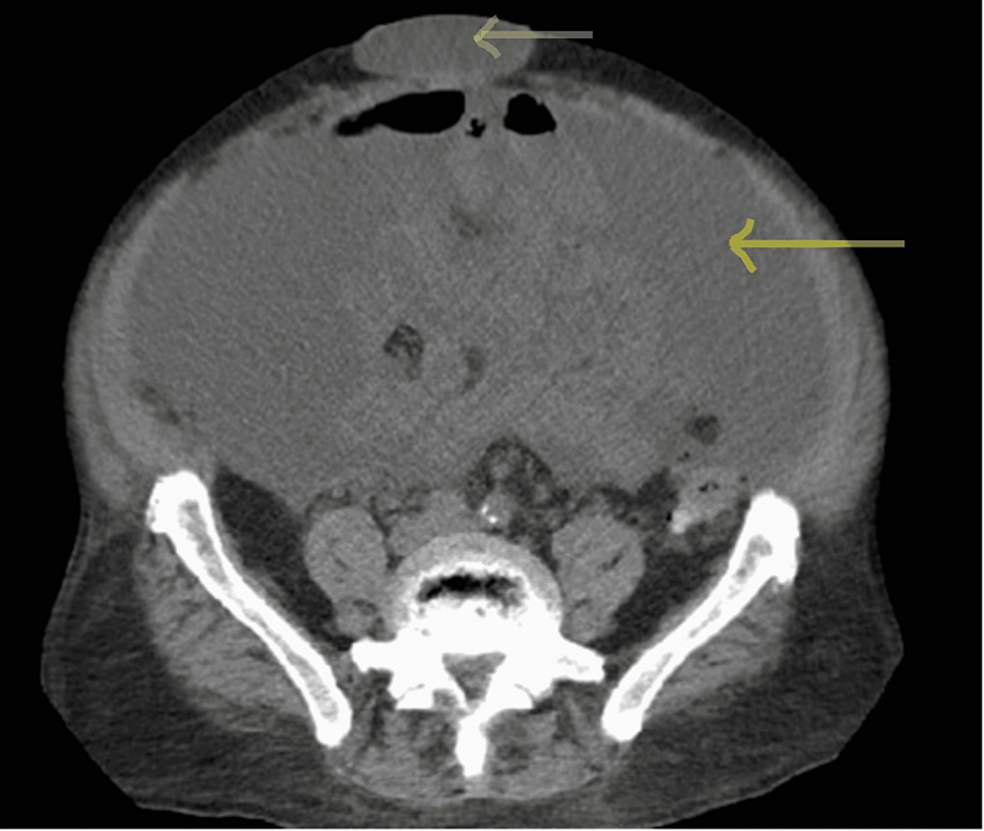
CT scan of the abdomen with IV contrast after paracentesis CT scan of the abdomen with IV contrast after abdominal paracentesis showing improved abdominal ascites (yellow arrow), and a new bowel loop inside the umbilical hernia (red arrow).

The patient was started on broad-spectrum IV antibiotics. He underwent ultrasound-guided paracentesis to relieve his abdominal distension, yielding 8.6 L straw color fluid that was later negative for spontaneous bacterial peritonitis (Table 1). The following day the patient developed increased central abdominal pain with multiple episodes of vomiting. Physical examination was remarkable for increased size and an erythematous firm umbilical hernia which was non-reducible. Emergent CT abdomen showed high-grade small bowel obstruction with a transition point in the umbilical hernia (Figure 2).

The surgical team was consulted and an emergent exploratory laparotomy was carried out. Findings included a 6 cm segment incarcerated loop of small bowel that appeared contused, but viable. Hernia repair without a surgical mesh was performed and a JP (Jackson-Pratt) drain was also placed. The patient underwent cystoscopy at the same time and only showed cystitis changes. 

The patient had a slow recovery after his surgery and needed repeat ultrasound-guided paracentesis after four days of his surgery due to increased abdominal distension. The drainage yielded 4.7 L straw color fluid and was again negative for spontaneous bacterial peritonitis. His urine culture was positive for vancomycin-resistant *Enterococcus faecium* and he was started on intravenous linezolid and completed a seven-day course. He was discharged on Day 9 with appropriate follow-ups. He had scheduled abdominal paracentesis every four weeks for recurrent large volume ascites. He did not have any evidence of umbilical hernia recurrence on the follow-ups. 

## Discussion

The prevalence of umbilical hernia in the adult population is about 2% [5] and it makes up about 5% of all hernias with minimal female predominance [6]. Acquired umbilical hernias in adults occur most often in obese patients, pregnant women, and cirrhotic patients with abdominal ascites [7]. In ascites, the increased intra-abdominal pressure from fluid accumulation pushes the umbilical ring open and leads to abdominal content herniation [8]. In addition, protein-calorie malnutrition in cirrhosis patients leads to ventral abdominal muscle wasting and therefore contributes to an increased incidence of umbilical hernia in patients with cirrhosis [9]. Incarceration and strangulation of umbilical hernia can happen spontaneously or secondary to coexisting intra-abdominal pathology such as intra-abdominal abscess [10] or decompression of ascetic fluid both medically and by paracentesis [3]. 

The patient in our case had a history of decompensated alcoholic cirrhosis with abdominal ascites refractory to medical therapy; the ascites were the main risk factor for the development of umbilical hernia in our patient. The presence of an uncomplicated umbilical hernia was observed on physical examination on admission and also on abdominal imaging. The patient underwent therapeutic and diagnostic abdominal paracentesis on admission which was thought to be the precipitating factor for umbilical hernia incarceration the next day. The development of worsening abdominal pain with the new physical examination findings of irreducible umbilical hernia with surrounding tenderness was concerning for acute abdominal pathology, especially the incarcerated or strangulated hernia which was then confirmed with abdominal imaging findings. The only secondary cause potentially leading to the development of umbilical hernia incarceration, in this case, was the abdominal paracentesis procedure that was performed the previous day. The most reasonable explanation for this is the presence of large volume ascites inside the abdominal cavity providing floatability of the bowel contents. However, with the rapid removal of ascites fluid by paracentesis, there is decreased tension on the umbilical hernia ring, with the subsequent decrease in diameter and trapping of hernia sac contents [11]. 

The development of incarcerated or strangulated umbilical hernia in patients with ascites was not limited to fluid removal by paracenteses; it was also reported after the resolution of ascites with medical management with diuretics [3]. However, incarceration usually occurs weeks to months after starting diuretics for medical management of ascites. More rapid incarceration of umbilical hernia was also reported after the resolution of abdominal ascites with the transjugular intrahepatic portosystemic shunt (TIPS) [12]. 

The definitive treatment of incarcerated or strangulated umbilical hernia in cirrhotic patients is always emergency surgical hernia repair. However, the management of uncomplicated umbilical hernia in cirrhotic patients with ascites remains uncertain. Previously, this was often treated conservatively given concerns of postoperative recurrence and the high prevalence of intraoperative complications [7]. Recently, a more aggressive approach with pre-operative optimization of nutrition in addition to pre-operative TIPS followed by surgical repair of umbilical hernia has shown lower morbidity rates compared with the conservative approach [11] [13]. 

## Conclusions

The development of umbilical hernia incarceration or strangulation following ascites drainage with abdominal paracentesis is a rare but serious complication with high rates of morbidity and mortality. Physicians should be aware of this complication and should have a low threshold to investigate for this known complication when new or worsening abdominal pain develops post paracentesis. In patients who have an umbilical hernia and develop recurrent large volume ascites requiring frequent abdominal paracentesis, we recommend early umbilical hernia repair to avoid this potential complication.
